# The impact of hydrostatic pressure on the result of physiological measurements in various coronary segments

**DOI:** 10.1007/s10554-020-01971-w

**Published:** 2020-08-17

**Authors:** Áron Üveges, Balázs Tar, Csaba Jenei, Dániel Czuriga, Zoltán Papp, Zoltán Csanádi, Zsolt Kőszegi

**Affiliations:** 1 Division of Cardiology, Department of Cardiology, Faculty of Medicine, University of Debrecen, Debrecen, Hungary; 2 III. Department of Internal Medicine, Szabolcs–Szatmár–Bereg County Hospitals and University Teaching Hospital, 68. Szent István Street, Nyíregyháza, 4400 Hungary; 3 Kálmán Laki Doctoral School of Biomedical and Clinical Sciences, University of Debrecen, Debrecen, Hungary; 4 Division of Clinical Physiology, Department of Cardiology, Faculty of Medicine, University of Debrecen, Debrecen, Hungary

**Keywords:** Hydrostatic pressure, FFR, Resting Pd/Pa, 3D analysis

## Abstract

The effect of hydrostatic pressure on physiological intracoronary measurements is usually ignored in the daily clinical practice. Our aim was to investigate this effect on Pd/Pa (distal/aortic pressure) and FFR (fractional flow reserve). 41 FFR measurements between 0.7 and 0.9 were selected. The difference in the height of the orifice and that of the sensor was defined in mm on the basis of 3D coronary reconstruction. Resting Pd/Pa and FFR were adjusted by subtracting the hydrostatic pressure gradient from the distal pressure. Height measurements were also performed from 2D lateral projections for each coronary segment (n = 305). In case of the LAD, each segment was located higher (proximal: − 13.69 ± 5.4; mid: − 46.13 ± 6.1; distal: − 56.80 ± 7.7 mm), whereas for the CX, each segment was lower (proximal: 14.98 ± 8.3; distal: 28.04 ± 6.3 mm) compared to the orifice. In case of the RCA, the distances from the orifice were much less (proximal: − 6.39 ± 2.9; mid: − 6.86 ± 7.0; distal: 17.95 ± 6.6 mm). The effect of these distances on pressure ratios at 100 Hgmm aortic pressure was between − 0.044 and 0.023. The correction for height differences changed the interpretation of the measurement (negative/positive result) in 5 (12%) and 11 (27%) cases for the FFR (cut-off value at 0.80) and the resting Pd/Pa (cut-off value at 0.92), respectively. The clinical implementation of hydrostatic pressure calculation should be considered during intracoronary pressure measurements. A correction for this parameter may become crucial in case of a borderline significant coronary artery stenosis, especially in distal coronary artery segments.

## Introduction

### Fractional flow reserve (FFR) and non-hyperemic pressure ratio (NHPR) measurements

According to current guidelines, the physiological measurement of coronary artery stenoses is recommended in chronic coronary syndrome. Nowadays, FFR is considered to be a standard method for the evaluation of myocardial ischemia and the likely advantage of revascularization [1–7].

FFR is calculated as the ratio of distal coronary artery pressure (Pd) and aortic pressure (Pa) during maximal hyperemia, usually induced by intracoronary or intravenous adenosine [8].

The accuracy of physiological intracoronary measurements is influenced by several factors. Pitfalls may originate from the preparation (calibration, equalization) or from the measurement itself (submaximal hyperemia, drifting, whipping, wedging). In addition, the role of hydrostatic pressure influenced by the position of the pressure wire sensor in relation to the orifice is usually ignored.

Non-hyperemic pressure ratios (NHPR) are measured at resting phase, without the induction of maximum hyperemia. The average distal-to-aortic pressure ratio at rest (resting Pd/Pa) and the instantaneous wave-free ratio (iFR) are the most important non-hyperemic parameters, their popularity results from the lack of need for adenosine. Prior clinical trials (DEFINE-FLAIR, IFR SWEDEHEART) had proven that the iFR method (pressure ratio measured in a diastolic time of minimum myocardial resistance) has a similar ability to guide coronary revascularization as FFR [9–11]. Previously, a close correlation between iFR and resting Pd/Pa had been shown [12–14]. The resting Pd/Pa value of 0.92 was defined as cut-off.

### Computed tomography versus invasive angiography

In recent years, studies investigating the effect of hydrostatic pressure on intracoronary indices have been published. Most of these examinations were performed using computed tomography (CT) angiographies to calculate the height differences between the orifice and the different segments of the coronary arteries [15–18]. Recently, an invasive angiography-based study has also highlighted the importance of height difference during pressure measurements at the highest and lowest sensor positions, potentially influencing FFR, iFR and Pd/Pa values. This study has challenged the concept of a single cut-off value for every coronary vessel [19].

Our aim was to investigate the effect of resting Pd/Pa and FFR adjustment based on the calculation of hydrostatic pressure gradient between the coronary orifice and the pressure wire sensor, to identify the relevance of hydrostatic pressure during clinical decision making, particularly in cases where FFR values were near the cut-off (between 0.7 and 0.9). We also aimed at specifying the effect of hydrostatic pressure in different segments of the coronary artery system.

## Methods

### Study design and population

This study was designed as a single center retrospective experiment to verify height differences between the coronary orifice and the pressure sensor, thereby exploring the impact of hydrostatic pressure. Analyses were performed based on two and three-dimensional methods in patients undergoing intracoronary pressure measurements for the assessment of intermediate coronary stenoses (50–90% diameter stenosis) from December 2016 to May 2019. The study complies with the Declaration of Helsinki; data were analyzed anonymously.

### Intracoronary pressure measurement

All catheterizations were performed using the radial approach. Following unfractionated heparin (5000 IU) administration, the pressure wire (PressureWire™ X Guidewire, Abbott) was positioned at the tip of the 6F guiding-catheter. Next, nitrate was administered and the pressures were equalized at the tip of the catheter. Then, the wire was advanced distally to the stenosis by 2–3 cm. FFR measurement was performed during hyperemia induced by intracoronary bolus of 200 μg adenosine. Pressure curves were recorded continuously until the hyperemic effect completely eliminated, and pressures reverted back to the resting Pd/Pa ratio. At the end of the procedure, the pressure sensor was pulled-back to the tip of the catheter to exclude any pressure drift.

### 3D reconstruction

A dedicated software package (QAngio^®^ XA 3D Research Edition 1.0 program, Medis Specials bv, Leiden, The Netherlands) was used for 3D coronary artery reconstruction from two angiographic views (at least with 25° difference). First, the program was calibrated (mm/pixel). As automatic calibration appeared to be a less repeatable method based on the first 17 cases, catheter calibration was used in our study thereafter, despite the fact that catheter calibration can be theoretically appropriate only when the spatial distribution of the coronary artery is close to the plane of the catheter. Reconstructions were performed during end-diastole by selecting the appropriate frame on the basis of the ECG traces. The amount of time needed to perform 3D analysis is approximately 3–4 min.

### Height difference measurement in 3D

**Fig. 1:**
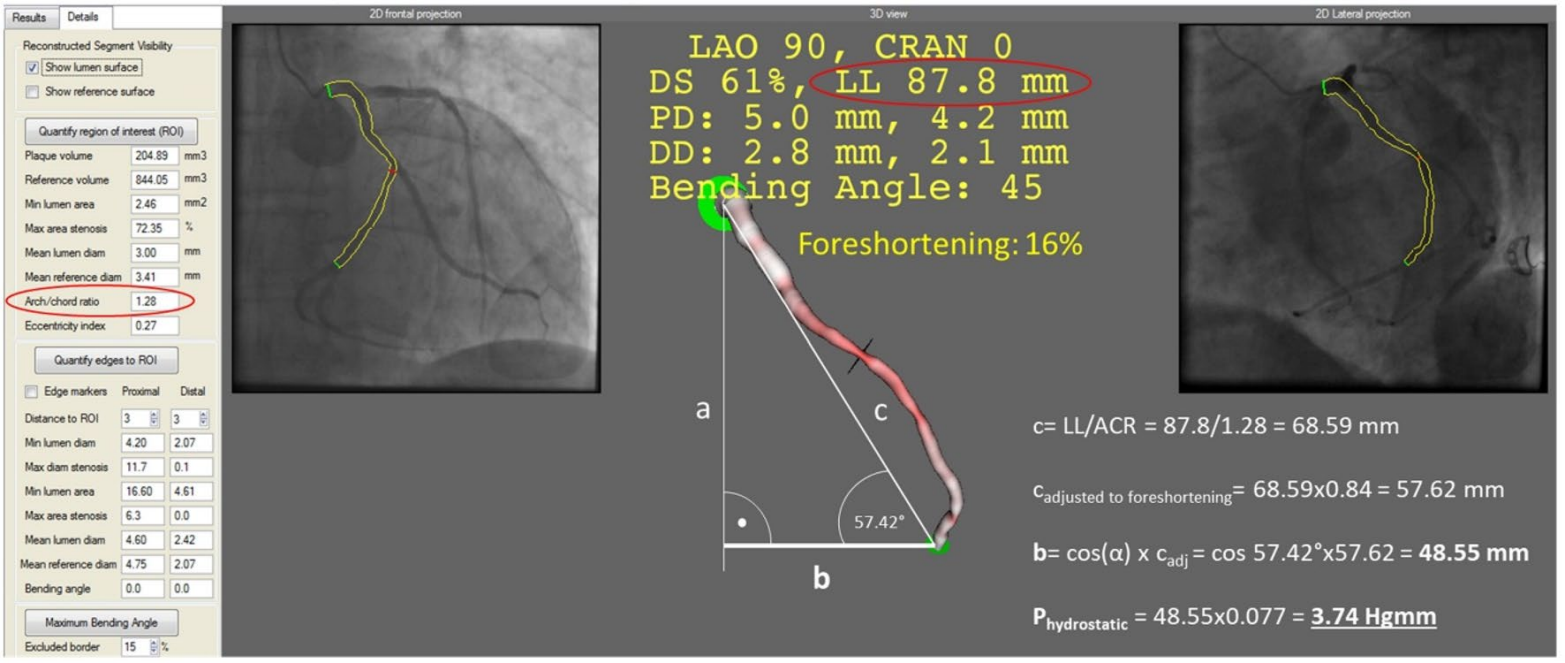
Height difference measurement in 3D. The final model included the length of the coronary artery segment (LL), the arc- chord ratio (arc as the midline of the analyzed segment; chord as the distance between the proximal and distal edges of the analyzed segment) and the foreshortening of the actual view. Following the correction of the chord length with the degree of foreshortening, a right triangle with a chord (c) as hypotenuse was created. Within this triangle, the cosines of the angle at the distal part (α) multiplied by the length of the chord (c) resulted in the height difference (b) between the orifice and the pressure wire sensor

After the 3D reconstruction, the coronary model was rotated to a lateral projection (LAO 90°, CAUD 0°). From this view, the height appeared without any foreshortening. The final model included the length of the coronary artery segment, the arc-chord ratio (arc as the midline of the analyzed segment; chord as the distance between the proximal and distal edges of the analyzed segment) and the foreshortening of the actual view [20]. Following the correction of the chord length with the degree of foreshortening, a right triangle with a chord as hypotenuse was created. Within this triangle, the cosines of angle at the distal part multiplied by the length of the chord resulted in the height difference between the orifice and the pressure wire sensor (Fig. 1).

### Height difference measurement in 2D

For 2D height detection, the quantitation software of the X-ray system was used on angiographic recordings acquired in the lateral view, where the height difference between the orifice and the sensor is projected without any foreshortening. In the patient’s supine position, the sternum is located on the left side of the screen, thus height differences are to be measured horizontally.

### Coronary segmentation defined by the Syntax nomenclature

A modified version of the coronary segmentation defined by the Syntax scoring system [21] was used in our study. This reproducible, schematic mapping of the coronary tree also accounting for the individual type of coronary circulation, creates an opportunity to determine the average height difference assigned to each coronary segment [22].

In our present study, ten epicardial coronary segments were evaluated. The left anterior descending artery (LAD) was divided into a proximal, a mid and a distal segment. The end-point of the proximal circumflex artery (CX) was defined at the origin of the obtuse marginal (OM) branch, while the distal CX corresponded to the distal run-off of the vessel. The main right coronary artery (RCA) was also divided into a proximal, a mid and a distal segment, while the posterolateral (PL) and posterior descending (PD) branches were evaluated separately. The end- points of the PL and PD branches were defined at the levels where the luminal diameter became less than 2 mm. Since coronary pressure measurements are generally not performed in the left main stem (LM; stenoses of the LM are usually analyzed by positioning the sensor in the proximal LAD or CX) or in small branches with a diameter less than 2 mm, these segments were not examined in our study. A feasible place of the pressure wire sensor was determined at the end of each coronary artery segment.

### Adjustment of hydrostatic pressure

The adjustment of resting Pd/Pa and FFR values was performed by adding 0.077 mmHg hydrostatic pressure per 1 mm height difference to the pressure measured in the distal coronary artery (Pd). This correction factor was based on the ratio of the specific gravity of mercury (13.55 g/cm^3^) and that of blood (1.05 g/cm^3^) [23].

### 2D and 3D measurements

In our study, we performed 3D reconstruction of 41 coronary lesions of 37 patients to assess the height difference between the catheter tip and the intracoronary pressure sensor. By this method, we were able to perform hydrostatic pressure calculations, even in lack of a lateral projection. We used this value to calculate the effect of the hydrostatic pressure on the measured resting Pd/Pa and on the FFR. In the next step, we investigated the correlation between the 3D height calculations and the 2D measurements carried out from the lateral view. Further, we measured the height difference between the catheter tip and preferably all ten segments predefined using the Syntax segmentation of the 37 patients from the lateral views of the coronary angiographies in 2D. Limited by the quality of the lateral views to determine the heights between the tip of the catheter and the most distal point of the segment in question, we performed 305 measurements in 2D.

### Statistical analyses

All analyses were performed using the Medcalc 12.2.1.0 program. Normality was assessed with normal probability (Q–Q) plot and with non-parametric Shapiro–Wilk test. All variables following normal distribution were compared using Student’s *t* test; for values not following normal distribution, the median and the interquartile range were expressed and compared between the groups using the Mann–Whitney *U* test. Continuous variables were reported as means with standard deviation (SD), while categorical variables were reported as numbers and percentages. Chi-squared test was performed for comparison of categorical variables. Clinical characteristics were analyzed per patient, lesion characteristics and pressure data per lesion. Significance level was defined as p < 0.05. The relation between 2 and 3D height difference measurements were assessed using a correlation analysis.

## Results

### Patient and lesion characteristics

During the examination period 147 FFR measurements were performed simultaneously with resting Pd/Pa detection. In case of 57 lesions, FFR values were between 0.7 and 0.9. In our patient population, ninety cases were out of this range with a percent diameter stenosis between 50 and 90. Sixteen cases were excluded due to incomplete hyperemia (caused by suboptimal cannulation of the orifice or developing a significant pause during the administration of adenosine), lack of a lateral DICOM view, poor image quality or images unsuitable for 3D reconstruction. Overall, 37 patients with 41 lesions were enrolled. The distribution of the lesions was the following: 3 proximal, 18 mid and 6 distal LAD, 1 proximal and 5 distal CX, 2 mid and 6 distal RCA. Hypertension, diabetes, dyslipidemia, age, body weight, height, body surface area (BSA, calculated from body weight and height), left ventricular end-diastolic diameter (LVEDD) and ejection fraction (EF) were examined, these data are presented in Table 1. Procedural results of the invasive physiological assessment, attributes of the investigated vessels (in terms of minimum lumen diameter of the interrogated lesion (MLD), percent diameter obstruction at MLD [%DS]), as well as resting Pd/Pa value and FFR value of the overall population are also presented in Table 1.

**Table 1:** Patient characteristics

Patient characteristics	All patients n = 37 (mean ± SD)	Female n = 16 (mean ± SD)	Male n = 21 (mean ± SD)	p value
Age	66.65 ± 6.22	68.06 ± 6.27	65.91 ± 6.74	0.3740
Weight (kg)	85.85 ± 16.47	77.73 ± 11.88	91.93 ± 15.28	0.0205*
Height (cm)	169.37 ± 6.75	163.40 ± 4.85	173.85 ± 6.75	**0.0002**
BSA (m^2^)	2.00 ± 0.22	1.87 ± 0.14	2.10 ± 0.19	0.0044*
LVEDD (mm)	55.36 ± 6.94	52.00 ± 6.40	57.76 ± 6.54	0.0504
EF (%)	50.89 ± 11.90	55.53 ± 12.69	47.57 ± 10.97	0.1136
Hypertension	35 (95.6%)	16 (100%)	19 (90.5%)	0.5923**
Diabetes	15 (40.5%)	6 (37.5%)	9 (42.9%)	0.7603**
Dyslipidaemia	17 (45.9%)	9 (56.3%)	8 (38.1%)	0.4444**
MLD (mm)	1.37 ± 0.34	1.34 ± 0.32	1.39 ± 0.35	0.7185
%DS	52.95 ± 6.28	53.13 ± 6.68	52.81 ± 5.97	0.9093
Resting Pd/Pa	0.90 ± 0.04	0.91 ± 0.05	0.89 ± 0.05	0.4498
FFR	0.83 ± 0.04	0.84 ± 0.03	0.82 ± 0.03	0.0765

Bold indicates statistical significance of p value < 0.05

*BSA* body surface area, *EF* ejection fraction, *FFR* fractional flow reserve, *LVEDD* left ventricular end-diastolic diameter, *MLD* minimum lumen diameter of the interrogated lesion, *resting Pd/Pa* distal-to-aortic pressure ratio at rest, *SD* standard deviation, *%DS* percent diameter obstruction at MLD

*Mann–Whitney test was performed on continuous variables showing non-normal distribution

**Chi-squared test was performed on categorical variables

### 3D reconstruction-based analysis of the coronary tree

The start point of the proximal LAD was usually located at a similar height as the left orifice. Overall, the LAD took an upward course with its highest point detectable at the left ventricular apex (distal LAD) in supine position. The overall CX ran in a downward course. The RCA took an upward course first, then the mid segment ran horizontally, and finally, in case of a right dominant coronary circulation, the distal RCA took a downward course and bifurcated into the PD and PL branches. The PD branch went towards the apex taking a slight upward course, while the direction of the PL branch was similar to that of the distal RCA.

### Correlation between 3 and 2D height differences between the catheter tip and the pressure wire sensor

**Fig. 2:**
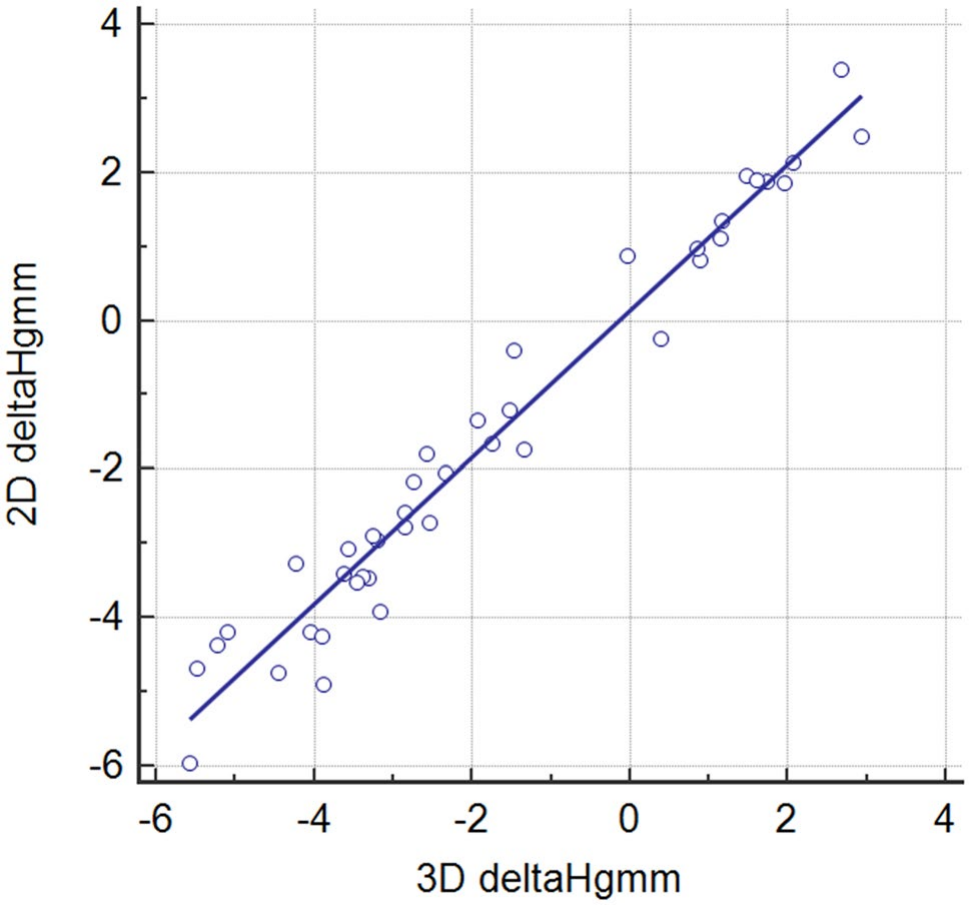
Correlation between the 3D and 2D methods of height measurement. A strong correlation was found between the 3D and 2D methods (r = 0.98; p < 0.001)

A 3D reconstruction rotated into the lateral projection was applied in order to determine the degree of height difference. The 2D quantitative coronary analysis (QCA) software of the local catheterization laboratory (Syngo Angio; Siemens) was used for simple distance measurements with an automated calibration from the lateral view (LAO 90°, CAUD 0°). We found a close correlation between the two methods (r = 0.9805, p < 0.0001; Fig. 2).

### Analysis of height difference between the catheter tip and different coronary artery segments based on the Syntax segmentation

The circulation type of the coronary anatomy was defined by assessing the 2D left and right coronary angiograms, as published in our previous paper [22]. This approach was used to provide a more accurate description of the 10 investigated coronary segments, compared to the Syntax score system. Three hundred and five measurements were performed using 2D lateral projections. The most distal point of the segment was compared to the tip of the catheter. In case of the LAD, every segment was located higher than the orifice (proximal LAD: − 13.69 ± 5.4 mm; mid LAD: − 46.13 ± 6.1 mm; distal LAD: − 56.80 ± 7.7 mm), and the highest point of the vessel was at the apex. The studied segments of the CX were located lower than the orifice (proximal CX: 14.98 ± 8.3 mm; distal CX: 28.04 ± 6.3 mm), while height differences measured for the RCA were least prominent (proximal RCA: − 6.39 ± 2.9 mm; mid RCA: − 6.86 ± 7.0 mm; distal RCA: 17.95 ± 6.6 mm). All studied PL and PD branches originated from the RCA, their height differences were 29.65 ± 6.1 and 17.53 ± 6.6 mm, respectively (Table 2).

**Table 2:** Influence of height differences on FFR and Pd/Pa values in ten coronary artery segments at 100 Hgmm aortic pressure

Coronary segment	Height differences (mm)	Hydrostatic pressure (Hgmm)	Influence of height differences on 0.8 FFR value at 100 Hgmm aortic pressure	Influence of height differences on 0.92 Pd/Pa value at 100 Hgmm aortic pressure	Delta FFR and Delta Pd/Pa
No height difference	0	0	0.8	0.92	0
Proximal LAD	− 13.69 ± 5.4	− 1.054 ± 0.41	0.811	0.931	− 0.011
Middle LAD	− 46.13 ± 6.1	− 3.552 ± 0.47	0.836	0.956	− 0.036
Distal LAD	− 56.80 ± 7.7	− 4.374 ± 0.59	0.844	0.964	− 0.044
Proximal CX	14.98 ± 8.3	1.153 ± 0.64	0.788	0.908	0.012
Distal CX	28.04 ± 6.3	2.159 ± 0.49	0.778	0.898	0.022
Proximal RCA	− 6.39 ± 2.9	− 0.492 ± 0.22	0.805	0.925	− 0.005
Middle RCA	− 6.86 ± 7.0	− 0.528 ± 0.54	0.805	0.925	− 0.005
Distal RCA	17.95 ± 6.6	1.382 ± 0.49	0.786	0.906	0.014
Posterolateral	29.65 ± 6.1	2.283 ± 0.47	0.777	0.897	0.023
Posterior descendent	17.53 ± 6.6	1.350 ± 0.50	0.787	0.907	0.014

*CX* circumflex artery, *FFR* fractional flow reserve, *LAD* left anterior descending coronary artery, *Pd/Pa* distal/aortic pressure, *RCA* right coronary artery

### Effect of hydrostatic pressure on FFR and resting Pd/Pa values per different coronary artery segments

**Fig. 3:**
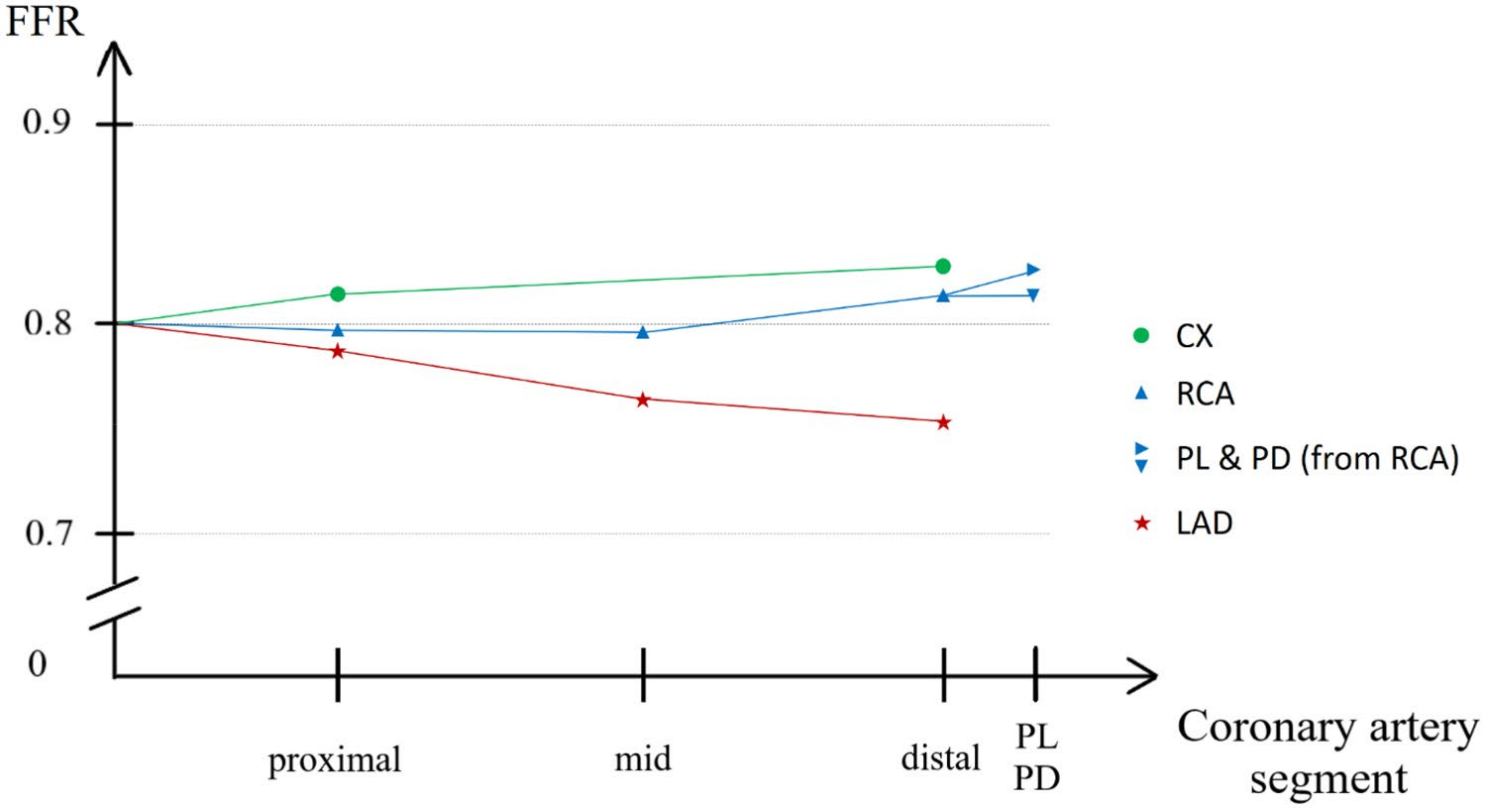
Conceptual scheme of the effect of hydrostatic pressure on the cut-off value of 0.80 FFR in different coronary artery segments. Invasively measurable pressure ratio in different coronary artery segments if the flow resistance results in a 0.80 FFR value in a particular segment. The hydrostatic pressure decreases the FFR value in the mid and distal LAD (left anterior descending artery), while there is an apparent increase in the distal CX (left circumflex artery). There is also a slight increase in the distal branches of the RCA (right coronary artery)

The effect of hydrostatic pressure on the cut-off value of 0.80 FFR in different coronary artery segments is summarized on the conceptual scheme of Fig. 3. The hydrostatic pressure decreased the FFR value in the mid and distal LAD, while there was an apparent increase in the distal CX.

In our study population (41 lesions with FFR measurements between 0.7 and 0.9), the correction for height differences changed the interpretation of the measurement (negative/positive result) in 5 (12%) and 11 (27%) cases for the FFR (cut-off value at 0.80) and the resting Pd/Pa (cut-off value at 0.92) measurements, respectively.

### Effect of body structure on height difference

Body structure influences the size of the heart, which corresponds to the distance between the coronary orifice and the coronary artery segments. The body weight, body height and therefore the body surface area (BSA) significantly affected the height differences measured between the coronary orifices and some epicardial segments. In our study, the body weight demonstrated a stronger correlation with the distances between the coronary orifices and the coronary artery segments than the body height, especially in case of the RCA. The impact of BSA was similar to that of the body weight (Table 3).

**Table 3:** Correlations between parameters describing body structure and height differences in corresponding coronary artery segments

Correlation	LADprox	LADmid	LADdist	Cxprox	Cxdist
Weight (kg)					
p	0.4879	0.2204	**0**.**0004**	0.9831	0.7666
r	− 0.1231	− 0.2157	− 0.5728	− 0.003785	− 0.05285
95% CI	− 0.4429 to 0.2244	− 0.5162 to 0.1321	− 0.7632 to − 0.2910	− 0.3415 to 0.3348	− 0.3842 to 0.2905
Height (cm)					
p	0.5676	0.4496	**0**.**0148**	0.9003	0.3322
r	− 0.1016	− 0.1341	− 0.4146	0.02233	0.1715
95% CI	− 0.4251 to 0.2450	− 0.4518 to 0.2138	− 0.6602 to − 0.08889	− 0.3182 to 0.3578	− 0.1770 to 0.4817
BSA (m^2^)					
p	0.4598	0.2005	**0**.**0003**	0.9935	0.9598
r	− 0.1311	− 0.2251	− 0.5857	− 0.001453	− 0.008973
95% CI	− 0.4494 to 0.2167	− 0.5235 to 0.1223	− 0.7712 to − 0.3087	− 0.3395 to 0.3369	− 0.3461 to 0.3302
LVEDD (mm)					
p	**0**.**0386**	**0**.**0066**	0.1209	0.2215	0.4129
r	− 0.3564	− 0.4571	− 0.2711	0.2152	0.1451
95% CI	− 0.6199 to − 0.02073	− 0.6888 to − 0.1406	− 0.5581 to 0.07379	− 0.1326 to 0.5159	− 0.2030 to 0.4607

Correlation	RCAprox	RCAmid	RCAdist	PL	PD
Weight (kg)					
p	0.3532	**0**.**014**	**0**.**001**	**0**.**0253**	**0**.**0068**
r	− 0.1859	− 0.4672	− 0.6277	− 0.4866	− 0.5715
95% CI	− 0.5286 to 0.2089	− 0.7195 to − 0.1060	− 0.8227 to − 0.3003	− 0.7589 to − 0.06951	− 0.8047 to − 0.1856
Height (cm)					
p	0.7879	0.1152	0.145	0.1079	0.1915
r	− 0.0543	− 0.3103	− 0.3066	− 0.361	− 0.2967
95% CI	− 0.4255 to 0.3326	− 0.6175 to 0.07906	− 0.6319 to 0.1104	− 0.6858 to 0.08375	− 0.6457 to 0.1548
BSA (m^2^)					
p	0.3688	**0**.**0117**	**0**.**0016**	**0**.**0257**	**0**.**0089**
r	− 0.1801	− 0.4778	− 0.609	− 0.4853	− 0.5561
95% CI	− 0.5242 to 0.2147	− 0.7260 to − 0.1195	− 0.8127 to − 0.2726	− 0.7582 to − 0.06787	− 0.7966 to − 0.1637
LVEDD (mm)					
p	0.1282	**0**.**0192**	0.2929	0.5279	0.7738
r	− 0.2945	− 0.4396	− 0.219	0.1422	0.065
95% CI	− 0.6015 to 0.08826	− 0.6982 to − 0.07959	− 0.5652 to 0.1928	− 0.2972 to 0.5319	− 0.3667 to 0.4736

*BSA* body surface area, *CX* circumflex artery, *LAD* left anterior descending coronary artery, *LVEDD* left ventricular end-diastolic diameter, *PD* posterior descending branch, *PL* posterolateral branch, *RCA* right coronary artery

The LVEDD measured by 2D echocardiography showed a significant correlation with the distance between both the left coronary orifice and the proximal and mid LAD, and the right coronary orifice and the mid RCA (Table 3).

## Discussion

According to Pascal´s law the hydrostatic pressure in the coronary arteries can be assumed as 0.77 mmHg per cm height difference, in case of a normal mass density (1050 kg/m^3^). Studies applying CT coronary angiography have previously showed that pressure differences are systematically detectable between the anterior and posterior coronary territories in supine position [15–18]. Moreover, height measurements at the highest or lowest points of the individual vessels indicated remarkable differences. In a previous study, intracoronary pressure measurements (resting Pd/Pa and FFR) were carried out in both supine and prone positions, and height differences were analyzed based on CT images [15]. These studies unequivocally found a significantly lower resting Pd/Pa and FFR values measured in the LAD, while higher values were demonstrated when measurements were carried out in the CX or RCA [15–18].

Hydrostatic pressure calculated from height difference measurements is a constant parameter. The effect of this parameter depends on general pressure conditions, being more prominent at lower pressures. The direction of the effect depends on the orientation of the sensor compared to the coronary orifice. Higher sensor positions result in increasing, while lower positions decreasing FFR and Pd/Pa values. During routine invasive coronary angiography, the determination of height differences between the coronary orifice and the pressure sensor using 2D or 3D assessment enables the correction of FFR and resting Pd/Pa ratios, by subtracting the hydrostatic pressure from the measured distal pressure. We have shown that hydrostatic pressure can be accurately calculated based on 3D coronary reconstruction, 2D data closely correlated with it.

In our study, the effect of the calculated hydrostatic pressure difference for ten epicardial coronary segments of the Syntax nomenclature were analyzed. We found a similar range of values as reported in prior studies, however, in the past, no specifications for the Syntax segmentation were available. For example, Harle et al. found a mean bias of FFR caused by hydrostatic pressure compared to a zero level of − 0.048 in the LAD, 0.02 in the CX, and 0.02 in the RCA. In our study, these values were − 0.011, − 0.036 and − 0.044 for the proximal, mid and distal segments of the LAD, respectively; while they were 0.012 and 0.022 for the proximal and distal CX, respectively. In case of the RCA, the average difference between the pressure ratios in the calculated and corrected values were − 0.005 for the proximal and mid, and 0.004 for the distal RCA, respectively. The change in the pressure ratios after the correction for hydrostatic pressure were 0.023 and 0.014 for the PL and PD branches, respectively.

**Fig. 4:**
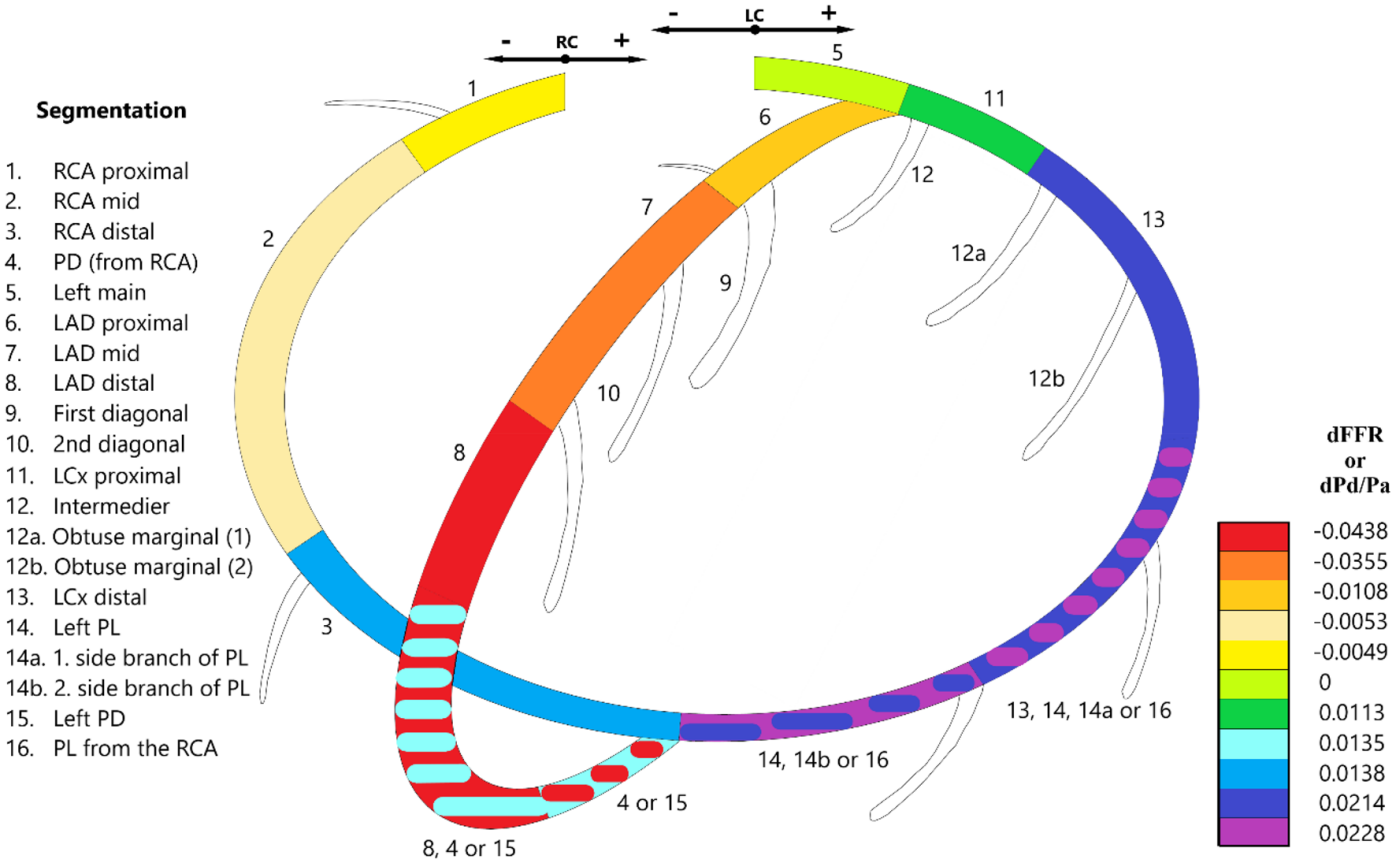
A modified color-coded version of the coronary segmentation defined by the Syntax scoring system. On this scheme, the change in fractional flow reserve and resting distal/aortic pressure values (dFFR and dPd/Pa) caused by hydrostatic pressure are indicated for each epicardial segment according to the type of the individual coronary circulation. Hydrostatic pressure was taken into account from the orifice of the right or left coronary artery (RC or LC)

When evaluating an individual coronary circulation, the variations in coronary anatomy need to be considered. The Syntax epicardial segmentation incorporates the Laeman classification with two main coronary circulation types [21]. However, it is known that the individual coronary anatomy may show further variations depending on the length of the LAD and the spatial distribution of the CX and RCA [24]. Of note, all PL and PD branches originated from the RCA in our study population. In one of our previous papers, we suggested an extension of the Syntax classification to the 12 different coronary patterns [25]. In our current study, we used a similar classification to interpret the results of the hydrostatic pressure measured in the individual coronary artery segments (Fig. 4). On this scheme, the change in FFR and resting Pd/Pa values are indicated for each epicardial segment according to the type of the individual coronary circulation. It was our team which used this approach first evaluating the role of hydrostatic pressure individually for each coronary artery segment.

Given the dichotomous interpretation of stenosis severity by the FFR measurement, we found a similar rate in the change of classification of an intermediate severity coronary artery stenosis after adjusting for hydrostatic pressure as in previous publications [15–18]. As a result of the correction of pressure ratios at 100 Hgmm aortic pressure, the interpretation of the measurements changed in 5 (12%) and 11 (27%) cases in our study population. This rate is in accordance with previous data (12.9%) [19], and overall represents the potential clinical significance of hydrostatic pressure measurement.

As body weight (and consequently the BSA) significantly influenced the measured height differences between the coronary orifices and most of the epicardial segments, normalization for this parameter may also be necessary in the future to create a universal correction factor for hydrostatic pressure. To this end, larger scale studies are needed to establish a well-defined normalized correction factor for each coronary artery segment in all types of coronary circulation.

## Limitations

For a standardized analysis, we used end-diastolic frames of the cardiac cycle for height difference measurements in both 2D and 3D. Of note, the motion of the coronary arteries could result in slightly different height measurements when assessed from other frames. This phenomenon could especially affect the RCA and the CX due to their vertical displacement during contractions.

The accuracy of 3D reconstruction depends on the selection of the least foreshortened projections at least 25° apart. A proper calibration of the program and well-designated reference points are also prerequisite of accurate results. Thus, the quality of the available recordings and the accuracy of the point selections were crucial in our study and could potentially influence our results.

The individual mass densities were not taken into consideration. The correction factor for distal pressure was based on an averaged mass density of 1050 kg/m^3^. The actual hematocrit values could have slightly modified our results.

## Conclusions

Our results suggest that the clinical implementation of hydrostatic pressure calculation should be considered in case of coronary artery stenoses of borderline significance, especially in case of intracoronary pressure measurements in distal coronary artery segments. The change in FFR and resting Pd/Pa values caused by hydrostatic pressure is inversely proportionate to the actual aortic pressure. The direction of the change depends on the vertical orientation of the sensor to the coronary orifice.

Hydrostatic pressure values measured in the same coronary artery segments from different patients were similar, however, larger scale studies are necessary to establish a well-defined correction factor for each coronary artery segment to enable an empirical, segment-based decision making.

Furthermore, in the era of image-based FFR (e.g. QFR), an accurate correction of the invasively measured pressure as a reference may improve algorithms to calculate pressure gradients and develop an accurate, less invasive assessment of coronary physiology.
